# Health and nutrition knowledge, attitudes and practices of pregnant women attending and not-attending ANC clinics in Western Kenya: a cross-sectional analysis

**DOI:** 10.1186/1471-2393-13-146

**Published:** 2013-07-11

**Authors:** Nandita Perumal, Donald C Cole, Hermann Z Ouédraogo, Kirimi Sindi, Cornelia Loechl, Jan Low, Carol Levin, Christine Kiria, Jaameeta Kurji, Mary Oyunga

**Affiliations:** 1 Dalla Lana School of Public Health, University of Toronto, Ontario, Canada; 2 International Potato Center, Nairobi, Kenya; 3 International Potato Center, Kampala, Uganda; 4 PATH, Seattle, Washington, USA; 5 Department of Global Health, University of Washington, Seattle, Washington,, USA; 6 Kenya Agriculture Research Institute (KARI), Kisumu, Kenya

**Keywords:** Knowledge, attitudes and practices (KAP), Developing countries, Antenatal care, Kenya

## Abstract

**Background:**

Antenatal care (ANC) is a key strategy to decreasing maternal mortality in low-resource settings. ANC clinics provide resources to improve nutrition and health knowledge and promote preventive health practices. We sought to compare the knowledge, attitude and practices (KAP) among women seeking and not-seeking ANC in rural Kenya.

**Methods:**

Data from a community-based cross-sectional survey conducted in Western Province, Kenya were used. Nutrition knowledge (NKS), health knowledge (HKS), attitude score (AS), and dietary diversity score (DDS) were constructed indices. χ^2^ test and Student’s t-test were used to compare proportions and means, respectively, to assess the difference in KAP among pregnant women attending and not-attending ANC clinics. Multiple regression analyses were used to assess the impact of the number of ANC visits (none, <4, ≥4) on knowledge and practice scores, adjusting for maternal socio-demographic confounders, such as age, gestational age, education level and household wealth index.

**Results:**

Among the 979 pregnant women in the survey, 59% had attended ANC clinics while 39% had not. The mean (±SD) NKS was 4.6 (1.9) out of 11, HKS was 6.2 (1.7) out of 12, DDS was 4.9 (1.4) out of 12, and AS was 7.4 (2.2) out of 10. Nutrition knowledge, attitudes, and DDS were not significantly different between ANC clinic attending and non-attending women. Among women who attended ANC clinics, 82.6% received malaria and/or antihelmintic treatment, compared to 29.6% of ANC clinic non-attendees. Higher number of ANC clinic visits and higher maternal education level were significantly positively associated with maternal health knowledge.

**Conclusions:**

Substantial opportunities exist for antenatal KAP improvement among women in Western Kenya, some of which could occur with greater ANC attendance. Further research is needed to understand multi-level factors that may affect maternal knowledge and practices.

## Background

The fifth Millennium Development Goal outlines the international commitment to measurably reduce maternal mortality by the year 2015 [1]. Antenatal care (ANC) is a critical strategy in reducing maternal mortality as it facilitates the identification and mitigation of risk factors early in pregnancy [2]. Timely and frequent use of ANC enables delivery of essential services, including malaria treatment, immunizations, and health counselling [3-6].

In particular, ANC clinics act as a key entry-point for implementing nutrition and health educational interventions that promote preventive health behaviours to improve maternal and neonatal health through better knowledge, attitudes and practices. Studies conducted in Turkey, Pakistan and Laos provide evidence to support the role of ANC in improving health knowledge, attitudes and practices (KAP) among women who utilize the service [7]. In the implementation and evaluation of a community-based ANC education programme in Istanbul [8], women in the ANC education group were reported to be more likely to initiate breastfeeding within the first two hours after delivery, bring infants for check-up within seven days after birth and to implement family planning measures at three months after birth, compared to the control group. Similarly, in a cross-sectional survey in Islamabad, Pakistan, Alam and colleagues showed that women attending ANC clinics were more likely to recognize signs of a difficult pregnancy, to realize the importance of eating a healthy diet, and to indicate tetanus immunization uptake, compared to their non-attending counterparts [9]. And in rural Laos, women who had received ANC were more likely to utilize health services at delivery and had a greater mean knowledge score regarding obstetric care compared to the women who had not received any antenatal care [10].

In Kenya, the World Health Organization (WHO) focused antenatal care guidelines, which recommend at least four antenatal visits for women with low-risk pregnancy and provide evidence-based content for each visit, were adopted and implemented in 2001 [11]. Despite a 92% national average for at least one ANC visit in 2008, the maternal mortality rate in Kenya remains at a high of 488 maternal deaths per 100,000 live births [12]. While this disparity may reflect challenges in feasibility and acceptability of the ANC package due to limited resources for materials and supplies, lack of training for service providers, and lack of clear policy direction [11], it may also be an indication of gaps in service provision at the ANC clinics, particularly in educational components. As Nikiema et al. showed in a cross-country analysis of Demographic Health Survey data from 19 sub-Saharan countries, healthcare providers do not routinely provide women with information as part of ANC or fail to provide information in a way that is remembered by women [13], which further impedes the efforts to improve health and nutrition KAP among women.

Among the few studies that assess the impact of ANC services on maternal KAP, few data emerge from sub-Saharan Africa. In a cross-sectional survey of women in rural Kenya, van Eijk et al. found that 11% of the women, who attended ANC clinics prior to delivery, were receptive to the health information provided during their visits [14]. In rural Nigeria women identified midwives and nurses as the major source of health information on maternal and child health during focus group discussions [15]. Yet, the comparative assessment of whether the KAP of women improved due to ANC utilization was not conducted in these studies. Recognizing the disparities in knowledge, attitudes, and practices of pregnant women who attend ANC clinics versus those who do not has important implications for assessing the impact of education interventions provided through ANC services.

The objective of this study, therefore, was to compare nutrition and health knowledge, attitudes, and practices of pregnant women who attended versus did not attend ANC clinics in rural Kenya. Specifically, we aimed to better understand the impact of ANC attendance on nutrition knowledge, health and healthcare knowledge, attitudes towards preventative health practices, and dietary diversity of pregnant women, taking into account other relevant covariates.

## Methods

### Study setting

The antenatal care package in Kenya follows the WHO evidence-based guidelines for comprehensive care and offers services such as weight and blood pressure measurement, tetanus toxiod vaccination, iron supplementation, tests for sexually transmitted infections, urinary glucose or protein, and HIV/AIDS, emergency preparedness and family planning, tuberculosis screening and detection, intermittent presumptive treatment (IPT) of malaria, and prevention of mother-to-child transmission of HIV. Additionally, the health education component of the ANC package includes counselling on birth planning, nutrition, physical activity during pregnancy, personal hygiene, and breastfeeding [11]. Pregnant women at low-risk of complications are recommended to attend ANC clinics for four comprehensive visits, starting in the first trimester of pregnancy (<16 weeks gestation) [11].

This study employs data from a community-based cross-sectional baseline survey undertaken between April-May, 2011, prior to the implementation of an innovative intervention that integrates orange-fleshed sweet potato (OFSP) promotion and production with nutrition education at ANC services in rural Kenya [16]. The baseline survey is a key part of the project evaluation strategy aimed at assessing the impact of integrating OFSP on the health and nutrition status of mothers and children less than two years of age. The survey was conducted in the catchment area of eight healthcare facilities serving a population of 362,151 individuals in the larger Bungoma and Busia districts of Western Province, Kenya [17]. The health facilities were selected based on the population served and the number of service providers, to facilitate the operational strategy of the intervention and to prevent leakage during OFSP project implementation.

### Data

A two-stage sampling strategy at the village and household level was used to select and recruit the target population of pregnant women and mothers with children 6–23 months old (mother-child pair). The sampling frame included all villages in the catchment areas of the selected health facilities with the number of households per village based on Kenya Census 2009 [17]. Out of 498 villages, 104 villages were selected according to a probability proportionate to size cluster sampling technique. In almost all villages (n = 97), a key informant interview was conducted with a village elder using a structured questionnaire. Key informants were asked about village characteristics, agricultural activities in their village, market access for produce, health services access, and the presence of village nutrition and health committees. On average there were 113 households per village. A within-village sampling frame was established through a door-to-door census of each village. Women who were pregnant or had an infant up to 23 months of age (or both) were invited to come to the survey site for informed consent and survey administration.

The survey questionnaires were administered by trained local enumerators. The survey team, composed of six enumerators and one supervisor, travelled to selected villages and established a survey site at a central location in the village. Eligible women were informed in advance of the survey location in the community, generally at a village elder’s homestead, and were interviewed by an enumerator upon arrival. A project car was provided for transport if women expressed difficulties in reaching the survey site. Prior to survey administration, participants provided informed consent in accordance with ethical clearance from the Kenya Medical Research Institute Research Ethics Board.

Each participant was asked a multi-dimensional questionnaire designed to collect data on several domains, including participant knowledge of nutrition, vitamin A and orange-fleshed sweet potato (OFSP), diet diversity and consumption of vitamin A rich foods, health seeking behaviours, child care practices, agricultural practices and socioeconomic factors. Field supervisors coordinated the survey and checked each questionnaire at the end of the field day to ensure optimal data quality. A total of 2761 women participated in the baseline survey, of which 1781 were mother-child pairs and 980 women were pregnant at the time of the survey. Only data from pregnant women were used for this analysis.

### Variables

#### *Outcome variables*

Four dependent variables characterizing maternal KAP were constructed; (1) nutrition knowledge score, (2) health & healthcare knowledge score, (3) attitude score, and (4) dietary diversity score.

Nutrition knowledge score (NKS) and health & healthcare knowledge score (HKS) were derived from key variables using equally-weighted summative item scores (see Additional file 1 for a list of survey items used). Weights were not used to generate knowledge scores as the selected items were relatively homogenous and equally important, and therefore, would not benefit from the added complexity of weighting and would risk incorrect weight assignment to items [18]. The NKS ranged from zero to eleven points and HKS ranged from zero to twelve points. The attitude score (AS) was also a summative score derived from Likert scale responses for hypothetical scenarios on health and nutrition. Participants’ responses to each of the hypothetical scenarios could range from ‘strongly agree’, ‘agree’, ‘neither agree or disagree’, ‘disagree’, or ‘strongly disagree’. A greater value was assigned to the most ideal response: ‘2’ for responses that reflected agreement with scenarios that show-cased preventative health practice or disagreement with scenarios that showed negative health-practices, ‘1’ for neutral responses and a score of ‘0’ for non-ideal responses. For instance, the scenario “*Emily feeds everyone in her household sweetpotato for breakfast because it is more nutritious than bread.*” seeks to assess the participants’ understanding of the nutritive value of foods. Agreement with this scenario would constitute an ideal response (i.e. score of ‘2’) and disagreement was regarded as non-ideal (i.e. score of ‘0’). Ideal and non-ideal responses for each scenario were judged by the research team. The AS ranged from zero to ten points.

The dietary diversity score (DDS) was the primary health practice of interest and was constructed from a 24-hour food recall, adding the number of different food groups out of twelve, which were consumed by the household within the last day [19]. Other health practices of interest were seeking treatment for malaria and intestinal worm (yes-no response for each) and qualitative assessment of amount of food intake during pregnancy as compared to before pregnancy (less than before, same as before, or greater than before).

#### *Independent variables*

**Figure 1:**
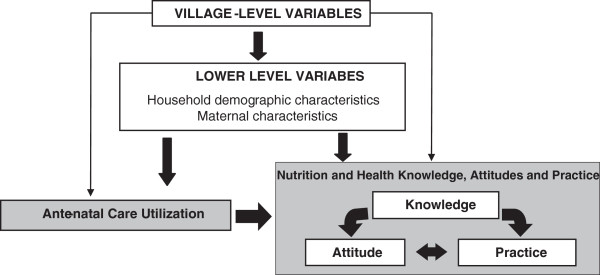
Conceptual model.

The primary independent variable was attendance at ANC clinic (yes vs. no) at the time of the survey. We conceptualized ANC clinics as a key resource for pregnant women to attain knowledge regarding health and nutrition. Based on our review of the literature and evidence from a systematic review of factors affecting the utilization of antenatal care in developing countries [20], we further identified maternal, household, and village-level factors that would confound the association between ANC utilization and nutrition and health KAP (Figure 1). Increased uptake and coverage of ANC has been previously associated with higher maternal age, lower parity, higher maternal educational level, and higher socio-economic status [7,14,21-23].

We used the conceptual framework to guide the variable selection for the adjusted analysis in this study. Maternal demographic and socioeconomic characteristics from the survey, including age, gestational age, marital status, education level, agricultural activity and selling agricultural products, involvement in income generating activities, and radio use, as a proxy for media interaction, were identified as possible confounders in the association between ANC utilization and dependent KAP outcomes. At the household-level, household head education level, household size, and socio-economic status were identified as potential confounders of ANC use. The wealth index was used as a proxy for household socio-economic status, derived by constructing a scale from household-quality variables, such as type of housing and roofing, presence and type of toilet, the source of cooking fuel and the source of water during dry season, in addition to possession of household assets such as radio, television, telephone/mobile, solar panels, gas cooker, bicycle, water pump, motorcycle, car truck, tractor, and generator. The wealth index was categorized into quartiles for descriptive analysis [24]. Community-level variables such as village size, the presence of village health and nutrition committees or other health interventions in the areas, and distance to the nearest health facility were recognized as factors that influenced the ability to seek ANC services and maternal KAP.

### Analysis

Data was entered using CSPro 4.0 software (US Census, 2011) and data cleaning was performed using SPSS version 19. Missing data and outliers were checked against hard-copy questionnaires to ensure accurate data entry. Data from 980 pregnant women who participated in the survey are included in these analyses. One case was excluded from further analyses due to a high proportion (>75%) of missing data. Descriptive statistics, including simple proportions, n (%), for categorical variables and mean with standard deviation for continuous variables, were noted for participant baseline characteristics.

#### *Unadjusted analysis*

In primary analysis, we hypothesized women who sought ANC services to demonstrate greater nutrition and health knowledge, positive attitudes towards preventive health practices, and better dietary diversity. Differences in knowledge (NKS and HKS), attitudes (AS) and dietary diversity (DDS) among women who had sought ANC services at least once at the time the survey versus those who had not, were assessed by Chi-square test and Student’s t-test, significant at two-sided alpha of less than 0.05.

#### *Adjusted analysis*

Due to the hierarchical nature of the data, multilevel modelling was initially employed to account for cluster sampling and to illustrate cross-village differences in the relationships between ANC attendance and maternal KAP [25,26]. NKS and HKS demonstrated intra-class correlations (ICCs) below 5%, indicating that the between-village variance explained less than 5% of the total variance in the two knowledge scores (see Additional file 2 for ICCs for all four dependent variables). This suggests that the inclusion of contextual variables in adjusted analyses would not add value to the model as village characteristics explained little variance in maternal knowledge. The DDS and AS demonstrated greater clustering by village with ICC values of 6.8% and 4.9%, respectively, demonstrating small effects of village-level independent variables. However, the village-level characteristics measured in the survey were similar between women who had attended ANC clinics compared to those who had not, providing little evidence for confounding due to these variables (see Additional file 3 for table comparing village-level characteristics). As an additional check, multi-level modelling conducted for the dependent outcomes did not change the inferences (data not shown).

Hence, multiple linear regressions were employed to assess the impact of ANC attendance (none, <4, ≥4 visits) on NKS, HKS, DDS and AS, controlling for maternal and household-level confounders. A forward selection model-building approach was used, whereby independent variables were excluded from the model if they were insignificant above a two-sided p-value of 0.10 and did not substantially change the beta-coefficients of other variables when excluded (<10% change). In the final models, statistical significance for all variables was set at p < 0.05. Several interaction terms were tested in the models and included if they were statistically significant. Model fit was assessed by adjusted R-squared for linear regressions [25]. All statistical analyses were conducted using SAS version 9.2.

## Results

Participant characteristics and demographic factors are presented in Table 1. Overall, 582 (59.4%) of surveyed women reported attending ANC clinics, while 382 (39%) of the women had not attended ANC clinics for their current pregnancy. Women who attended ANC clinics were on average two months greater in gestational age compared to non-attendees, with the mean first ANC visit at 4.5 months of pregnancy. A greater proportion of ANC seeking women were single (12.7%) compared to non-attending women (5.7%).

**Table 1:** Participant and household demographics by whole sample and ANC attendance

**Variable**	**Overall**	**ANC attendance**^**a**^		p-values*
		**Attending**	**Non-attending**	
n (%)	979	582 (59.4)	382 (39.0)	
Age, years	26.38 ± 6.6	26.20 ± 6.8	26.72 ± 6.4	0.233
Average gestational age, months^a^	5.88 ± 2.1^1^	6.97 ± 1.6	4.25 ± 1.6	<0.0001
Gestational age at first ANC visit, months^a^	4.48 ± 1.5^1^	4.48 ± 1.5	-	-
Marital status, n (%)				
Single	97 (9.9)	74 (12.7)	22 (5.7)	0.001
Married	868 (88.6)	497 (85.4)	358 (93.8)	
Divorced/separated	14 (1.43)	11 (1.9)	3 (0.78)	
Household size	4.65 ± 2.1	4.60 ± 2.2	4.71 ± 2.1	0.478
Household head, n (%)^b^				
Male headed	905 (92.9)	526 (91.0)	365 (95.8)	0.002
Female with male support	43 (4.4)	28 (4.8)	14 (3.7)	
Female without male support	26 (2.7)	24 (4.2)	2 (0.5)	
Household head education level^c^				
Less than primary	307 (31.4)	176 (30.2)	124 (32.5)	0.041
Primary school complete	278 (28.4)	151 (26.0)	124 (32.5)	
Above primary school	387 (39.5)	250 (43.0)	132 (34.6)	
Mother’s education level^c^				
Less than primary	393 (40.4)	218 (37.5)	169 (44.1)	0.121
Primary school complete	324 (33.1)	201 (34.6)	118 (30.8)	
Above primary school	261 (26.7)	162 (27.9)	96 (25.1)	
Agricultural activity and selling agriculture products				
Not involved in agriculture/selling products	63 (6.4)	48 (8.3)	14 (3.7)	0.004
Secondary activity, not selling agri products	81 (8.3)	55 (9.5)	25 (6.5)	
Secondary activity and selling products	145 (14.8)	87 (14.5)	56 (14.6)	
Principal activity, not selling agri. products	551 (56.3)	303 (52.1)	240 (62.7)	
Principal activity and selling products	139 (14.2)	89 (15.3)	48 (12.5)	
Salaried employment, yes	55 (5.6)	33 (5.7)	22 (5.7)	0.954
Casual labour, yes	384 (39.2)	199 (34.2)	175 (45.7)	<0.001
Involved in informal business, yes	196 (20.2)	124 (21.3)	70 (18.3)	0.259
Self-employment	224 (22.9)	117 (20.1)	104 (27.2)	0.012
Income generating activities^d^				
Not involved	315 (32.1)	200 (34.4)	111 (28.9)	0.003
Involved in one activity	486 (49.6)	297 (51.0)	181 (47.3)	
Involved in two activities	165 (16.8)	79 (13.6)	83 (21.7)	
Involved in three activities	14 (1.4)	6 (1.0)	8 (2.1)	
Wealth index^1,e^, quartiles				
1^st^ quartile (Lowest)	233 (24.2)	124 (21.3)	109 (28.5)	0.001
2^nd^ quartile	243 (25.2)	136 (23.4)	107 (28.0)	
3^rd^ quartile	240 (24.9)	150 (25.8)	90 (23.6)	
4^th^ quartile (Highest)	248 (25.7)	172 (29.6)	76 (19.9)	
Radio use				
Everyday	644 (65.8)	398 (68.4)	238 (62.3)	0.004
At least once per week	128 (13.1)	83 (14.3)	44 (11.5)	
Irregularly	112 (11.4)	60 (10.3)	50 (13.1)	
Do not listen	95 (9.7)	41 (7.0)	50 (13.1)	

^1^ Mean ± SD.

*p-values for Student’s t-test and chi-square test for comparison of means and proportions, respectively, between ANC attending and non-attending women, significant at a two-sided alpha of < 0.05.

^a^ Missing ANC attendance data for n = 15 women and gestational age data for n = 7 women; ^b^Missing values for household head, n = 5; ^c^Missing household head education level for n = 7 cases; effective n = 972 and missing values for highest level of education, n = 1 mother.

^d^ Income generating activities is a summative variable combining involvement in salaried employment, casual labour, informal business, and self-employment. None of the participants were involved in all four activities.

^e^ Missing data for n = 15 for wealth index status by ANC attendance.

Agriculture as a primary activity and selling agricultural products were significantly greater among women who sought ANC, compared to women who did not attend ANC clinics (15.3% vs. 12.5%). Women who did not attend ANC clinics were more likely to be involved in agriculture as their primary activity but did not sell agricultural products (62.7% vs. 52.1%), were more likely to undertake casual labour (45.7% vs. 34.2%) and were more likely to be self-employed (27.2% vs. 20.1%) compared to ANC clinic attendees. The proportion of women involved in two or more income generating activities was greater among women who did not attend ANC clinics compared to ANC seeking women (21.7% vs. 13.6%). Conversely, a greater proportion of women who attended ANC clinics were either not involved in income generating activities or were involved in only one activity. In addition, household head education above primary school, wealth index and frequent radio use were significantly greater for ANC clinic attendees compared to non-attending women. Women who attended ANC clinics were less likely to be in the lowest wealth index quartile (21.3% versus 28.5%) and were more likely to listen to the radio every day or at least once a week compared to women who had not attended ANC clinic.

At the community level, there were no statistically significant differences between women who attended and did not attend ANC clinics. Village size ranged from 38 to 296 households and the number of pregnant women participating from villages ranged from two to 25 women; 72.5% of the women lived in villages which had previously held health meetings organized by The AIDS, Population and Health Integrated Assistance (APHIA) program; village committees for health and nutrition existed for 69.8% of the women; and health interventions had previously occurred in villages for 50.8% of the women.

Table 2 summarizes the unadjusted comparisons in nutrition and health knowledge scores, attitude score, and dietary diversity score of women by ANC attendance. Overall, 46% of the women in the study had a moderate level of nutrition knowledge, 44.6% had moderate health knowledge level, and 80.7% had moderate DDS. Attitude score was high (>7 out of 10) for 59.6% of the women. Health knowledge score was marginally higher among women who had attended ANC clinic compared to non-attendees (6.3 vs. 6.0, p = 0.045). Receiving malaria treatment alone and receiving malaria and anthelmintic treatment were significantly greater among women who had attended ANC. In adjusted analyses, the number of ANC clinic visits was significantly associated with health knowledge score, controlling for maternal age, education level, and involvement in income generating activities (Table 3). Compared to no ANC clinic visits, <4 ANC clinic visits were positively associated with greater health knowledge (0.35, p < 0.05), with the magnitude of the association increasing significantly for ≥4 ANC clinic visits (0.89, p < 0.001). In unadjusted and adjusted analyses, there were no significant differences in nutrition knowledge, attitudes, and dietary diversity of women based on ANC clinic attendance. Nonetheless, incremental increases in nutrition knowledge and health knowledge scores were predictive of positive health attitudes (0.09, p < 0.05 and 0.15, p < 0.01, respectively), adjusting for covariates. The amount of food consumption also did not differ statistically between the two groups; however, overall, women in both groups reported eating less during their pregnancy as compared to before pregnancy.

**Table 2:** Knowledge, attitudes and practices of pregnant women, overall and by ANC attendance

**Variable**	**Overall**	**ANC attendance**^**a**^		p-values*
		**Attending**	**Non-attending**	
n (%)	979	582 (59.4)	382 (39.0)	
Nutrition knowledge score	4.61 ± 1.9^1^	4.62 ± 1.9^1^	4.59 ± 1.9	0.839
Low (<4)	320 (32.7)	192 (32.9)	122 (31.9)	0.866
Moderate (4–6)	450 (46.0)	264 (45.4)	180 (47.1)	
High (7–11)	209 (21.4)	126 (21.7)	80 (20.9)	
Health and healthcare knowledge score	6.18 ± 1.7	6.28 ± 1.6	6.04 ± 1.8	0.045
Low (<5)	332 (33.9)	186 (31.9)	141 (36.9)	0.261
Moderate (6–7)	437 (44.6)	265 (45.5)	165 (43.2)	
High (8–12)	210 (21.5)	131 (22.5)	76 (19.9)	
Attitude score	7.39 ± 2.2	7.48 ± 2.2	7.24 ± 2.2	0.089
Low (<5)	138 (14.1)	74 (12.7)	62 (16.2)	0.259
Medium (5–7)	258 (26.4)	152 (26.1)	102 (26.7)	
High (8–10)	583 (59.6)	356 (61.2)	218 (57.1)	
Dietary diversity score	4.89 ± 1.4	4.90 ± 1.5	4.87 ± 1.4	0.803
Low(<4)	151 (15.4)	94 (16.2)	56 (14.7)	0.677
Moderate (4–7)	790 (80.7)	464 (79.7)	313 (81.9)	
High (8–12)	38 (3.9)	24 (4.1)	13 (3.4)	
Seeking malaria & antihelmintic treatment^b^				
No treatment	296 (34.4)	101 (17.4)	195 (70.4)	<0.0001^b^
Anthelmintic treatment alone	18 (2.1)	16 (2.8)	2 (0.7)	
Malaria treatment alone	386 (44.9)	321 (55.2)	65 (23.5)	
Both treatments	160 (18.6)	144 (24.7)	15 (5.4)	
Food intake during pregnancy^c^				
Less than before	419 (47.6)	272 (47.8)	145 (46.9)	0.266
Same as before	234 (26.6)	159 (27.9)	75 (24.3)	
More than before	228 (25.9)	138 (24.3)	89 (28.8)	

*p-values for Chi-square test for comparison proportions is significant at a two-sided alpha of <0.05.

^1^ Mean ± Standard Deviation (SD) for continuous scale; the scores are categorized for descriptive purposes.

^a^ Missing ANC attendance data for n = 15 women, effecting sample size for ANC attendance comparisons, n = 964.

^b^ Missing seeking treatment values for n = 120; effective n = 859; For chi-square statistic, antihelmintic treatment was combined with malaria treatment (seeking only one treatment) due to a cell count of <5.

^c^ Missing values for amount food intake during pregnancy, as compared to before pregnancy in n = 101 women; effective n = 878.

**Table 3:** Effect of antenatal care visits on knowledge, attitude and practice scores among pregnant women in rural Kenya, adjusted for covariates

**Characteristics**	**Health knowledge**^**1§**^		**Nutrition knowledge**^**2§**^		**Preventive attitude score**^**3§**^		**Dietary diversity score**^**4§**^	
	**Un-adjusted**	**Adjusted**	**Un-adjusted**	**Adjusted**	**Un-adjusted**	**Adjusted**	**Un-adjusted**	**Adjusted**
	**β (SE)**	**β (SE)**	**β (SE)**	**β (SE)**	**β (SE)**	**β (SE)**	**β (SE)**	**β (SE)**
Intercept	-	4.66 (0.30)	-	2.124 (0.33)	-	5.90 (0.38)	-	3.65 (0.37)
Age, yrs	0.04 (0.01)^***^	0.025 (0.01)^**^	0.04 (0.01)^***^	0.04 (0.01)^***^	0.0 (0.01)	-	-0.01 (0.01)	-0.02 (0.01)^**^
Number of ANC clinic visits								
None	Reference	Reference	Reference	Reference	Reference	Reference	Reference	Reference
<4 visits	0.12 (0.12)	0.35 (0.14)^*^	0.04 (0.13)	-0.17 (0.14)	0.26 (0.15)	0.23 (0.15)	-0.04 (0.10)	-0.09 (0.10)
≥4 visits	0.57 (0.18)^**^	0.89 (0.21)^***^	-0.03 (0.20)	-0.11 (0.21)	0.27 (0.23)	0.14 (0.22)	0.20 (0.15)	0.26 (0.14)
Gestational age								
1^st^ trimester	Reference	Reference	Reference	Reference	Reference	-	Reference	-
2^nd^ trimester	-0.21 (0.16)	-0.34 (0.17)^*^	0.29 (0.18)	0.32 (0.17)	0.01 (0.21)	-	0.06 (0.13)	**-**
3^rd^ trimester	-0.18 (0.16)	-0.52 (0.19)^**^	0.11 (0.18)	0.31 (0.19)	0.03 (0.20)	-	0.01 (0.13)	**-**
Mother’s education level								
Less than primary	Reference	Reference	Reference	Reference	Reference	Reference	Reference	Reference
Primary school complete	0.26 (0.13)^*^	0.26 (0.13)^*^	0.83 (0.13)^***^	0.821 (0.13)^**^	0.21 (0.16)	0.03 (0.16)	0.21 (0.11)^*^	0.15 (0.10)
Above primary school	0.57 (0.14)^***^	0.51 (0.14)^**^	2.26 (0.14)^***^	2.132 (0.14)^**^	0.83 (0.17)^***^	0.43 (0.19)^*^	0.52 (0.11)^***^	0.40 (0.12)^**^
Marital status								
Single	Reference	Reference	Reference	-	Reference	-	-	-
Married	0.68 (0.18)^**^	0.26 (0.20)	0.08 (0.21)	-	-0.14 (0.23)	-	-	-
Separated/Divorced	-0.38 (0.49)	-0.75 (0.48)	-0.70 (0.55)	-	-0.88 (0.62)	-	-	-
Wealth index	1.91 (0.83)^*^	0.88 (0.88)	4.05 (0.93)^***^	-0.13 (0.87)	0.98 (1.05)	-0.85 (1.09)	4.60 (0.67)^***^	3.90 (0.71)^***^
Agricultural activity and selling agriculture products								
Not involved in agriculture/selling products	Reference	-	Reference	-	Reference	Reference	Reference	Reference
Secondary activity, not selling agri products	0.66 (0.29)^*^	-	0.16 (0.32)	-	0.38 (0.38)	0.20 (0.36)	0.22 (0.24)	0.20 (0.24)
Principal activity, not selling agri. products	0.74 (0.23)^**^	-	0.06 (0.25)	-	-0.13 (0.28)	-0.21 (0.28)	-0.10 (0.19)	0.16 (0.19)
Secondary activity and selling products	0.65 (0.26)^*^	-	0.18 (0.28)	-	1.1 (0.32)^**^	0.93 (0.32)^**^	-0.49 (0.21)^*^	-0.24 (0.21)
Principal activity and selling products	0.44 (0.26)	-	-0.35 (0.29)	-	-0.42 (0.32)	-0.45 (0.33)	-0.45 (0.21)^*^	-0.19 (0.21)
Income generating activities								
Not involved	Reference	Reference	Reference	-	Reference	-	Reference	Reference
Involved in one activity	0.26 (0.12)^*^	0.17 (0.12)	0.02 (0.14)	-	-0.04 (0.16)	-	0.10 (0.10)	0.19 (0.10)
Involved in two activities	046 (0.17)^**^	0.37 (0.17)^*^	0.16 (0.19)	-	-0.09 (0.21)	-	0.51 (0.14)^**^	0.64 (0.14)^***^
Involved in three activities	0.67 (0.47)	0.32 (0.46)	1.00 (0.53)	-	-0.28 (0.60)	-	0.84 (0.39)^*^	0.77 (0.37)^*^
Radio use								
Do not listen	Reference	Reference	Reference	Reference	Reference	-	Reference	Reference
Irregularly	-0.31 (0.24)	-0.36 (0.24)	-0.22 (0.27)	-0.25 (0.24)	0.19 (0.30)	-	0.77 (0.20)^***^	0.58 (0.20)^*^
At least once per week	-0.00 (0.23)	-0.07 (0.23)	-0.31 (0.26)	-0.21 (0.23)	0.35 (0.30)	-	0.33 (0.19)	0.33 (0.19)
Everyday	0.42 (0.19)^*^	0.19 (0.19)	0.45 (0.21)^*^	0.21 (0.19)	0.02 (0.24)	-	0.65 (0.15)^***^	0.46 (0.16)^*^
Nutrition knowledge	-	-	-	-	0.17 (0.04)^***^	0.09 (0.04)^*^		
Health knowledge	-	-	-	-	0.18 (0.04)^***^	0.14 (0.04)^**^	-0.02 (0.03)	-0.05 (0.03)
Attitude score	-	-	-	-	-	-	0.01 (0.02)	-

§Unadjusted model signify the bivariate association between dependent and independent variables alone. Whereas, adjusted analysis is a reduces model taking into account all covariates that showed a significant association.

^1^ n = 23 observations excluded in the analysis due to missing values in ANC attendance (n = 15), gestational age (n = 7), and maternal education (n = 1); R^2^ = 0.08.

^2^ n = 23 excluded from analysis due to missing values in ANC attendance (n = 15), gestational age (n = 7), and maternal education (n = 1); R^2^ = 0.26.

^3^ n = 24 values not used in regression analysis due to missing values in ANC attendance (n = 15), gestational age (n = 7), maternal education (n = 1), and health knowledge score (n = 1); R^2^ = 0.10.

^4^ n = 24 observations excluded in the analysis due to missing values in ANC attendance (n = 15), gestational age (n = 7), maternal education (n = 1), and health knowledge score (n = 1); R^2^ = 0.12.

*** p significant at a two-sided alpha of <0.0001; **p-value significant at a two-sided alpha of <0.01; *p-value significant at a two-sided alpha of <0.05.

## Discussion

This study provides key insights into the impact of ANC attendance on the knowledge, attitudes and practices of pregnant women in rural Kenya. Among women who had attended ANC clinic at least once, health knowledge score and proportion who received malaria or anthelminthic treatment were significantly greater compared to their non-attending counterparts. Similar findings were observed in rural Pakistan and Laos where ANC clinic attendees demonstrated greater knowledge of obstetric complications than non-attending women [9,10]. Further, the positive association observed between the number of ANC clinic visits and higher health knowledge score, after controlling for maternal socio-demographic characteristics, is encouraging. This parallels the positive trend observed in the cross-country analysis of 19 sub-Saharan countries, where compared to women who had attended ANC clinics once, the likelihood of receiving advice was almost twice as much for women who attended ANC clinics three times, and closer to three times as much for women who visited ANC clinics at least five times [13]. This suggests that health and healthcare education provided at the ANC clinics may confer substantive improvements in health knowledge among pregnant women in rural Kenya.

Nonetheless, greater efforts to improve the quality and uptake of educational interventions implemented through ANC services are necessary in sub-Saharan Africa. The lack of difference in nutrition knowledge and dietary diversity outcomes by ANC clinic attendance observed in this study may be a result of homogenization of the maternal KAP due to village nutrition committees in the communities surveyed, limited uptake of information offered at ANC clinics, and/or due to a lack of effective nutrition counselling during ANC visits contributing to minimal nutrition knowledge gain [13]. Sub-optimal nutrition counselling has previously been observed in an acceptability and sustainability analysis of the focused ANC package in Kenya where only one third of the women attending ANC clinics for the first time received nutritional counselling [11]. In rural Uganda, ineffective organization of educational sessions recommended as part of the ANC service provision was noted as a major impediment to increasing maternal knowledge [27], and in The Gambia, Anya et al. showed that despite high rates of ANC attendance, there was limited benefit observed for women who sought ANC services [28]. Moreover, only 14% of the women surveyed in a previous cross-sectional survey conducted in Western Kenya reportedly attended a health talk at the ANC clinic and the health topics covered during ANC visits were noted to be limited in scope [14]. Similarly, in rural Tanzania, delivery of health information, including nutrition counselling, at the ANC clinics was noted to be among the least likely components of ANC to be effectively implemented [29]. These data indicate persistent gaps in effective counselling, specifically in nutrition topics, provided through ANC clinics.

Not surprising then, the average household dietary diversity score for women in this survey was less than five, a level previously demonstrated to correlate with micronutrient inadequacy [19]. Additionally, the reported lack of adequate food consumption during pregnancy, although not unique to the Kenyan context, is concerning. In a cross-sectional survey in rural Bangladesh, only one-third of women surveyed reported increasing their food intake during pregnancy [30], and in a focus-group analysis in Bali, women reported restricting the amount of food consumed during pregnancy to prevent having a large infant and a difficult delivery [31]. Such practices fail to meet the increased nutritional demands necessary to maintain optimal health during pregnancy [32] and undermine public health efforts targeted to improve maternal and peri-natal health.

In addition to ANC attendance, maternal education level was positively associated with health and nutrition knowledge, health attitudes, and dietary diversity. Higher maternal education level has been previously correlated with greater health knowledge scores among women in Laos, China and Turkey [8,10,33]. Gestational age, however, was negatively associated with health knowledge score in this population suggesting that a delay in seeking ANC may contribute to less health knowledge as the opportunities to receive advice and education are reduced. Radio use was also noted to be greater among ANC attending women and demonstrated positive associations with dietary diversity score, indicating that radio-broadcasted public health messages may be an effective method for providing educational programs to improve healthcare knowledge in this population. Media interaction, in the form of radio messages regarding health during pregnancy, has been reported to increase awareness among Guatemalan mothers and supplement their clinic-based education [34].

An interesting finding of this study was the positive association between income generating activities and health knowledge score. Although our results parallel those from China [12], where employed women were noted to have higher health knowledge compared to unemployed women, higher involvement in income generating activities was negatively associated with ANC attendance. In this study, a lower proportion of women involved in two income generating activities attended ANC clinics compared to women involved in only one activity or less. This finding provides evidence for the maternal “double-burden”, characterized by managing the increasing demands of labour and economic productivity with care-taking responsibilities [35]. Women who were involved in two income generating activities may have limited time to seek preventative ANC in this population.

Our interpretation regarding the temporality of the observed associations is limited due to the cross-sectional nature of the survey. In addition, although women were notified in advance of the survey date and transportation was provided to those who had difficulties reaching the survey site, inviting women to a centralized survey location in the village may have introduced selection bias and may limit the generalizability of these results. Also, although maternal parity is an important factor in antenatal care use, it was not possible to control for parity in adjusted analysis as this data was not directly collected in the survey. Women’s autonomy, gender relationships and social networks have been previously found to lend to differences in beliefs and preferences relating to maternal healthcare seeking behaviours [36], but were not explored in this analysis given the lack of heterogeneity in the data. Furthermore, although the use of a 24-hour recall to construct the DDS does not account for within-person variance, the DDS is considered to be a valid proxy indicator to reflect nutrition adequacy and is often employed in low resource settings [37]. Future studies should explore the factors associated with maternal health care knowledge and behaviours and utilize qualitative research to better understand the nuances in maternal KAP due to contextual variables.

## Conclusions

This study revealed that health knowledge and receiving malaria and anthelmintic practices alone were higher among women who attended ANC clinics compared to those who did not. Attending ANC clinics confers some benefit, but not in nutrition education, dietary diversity or improved food consumption practices. Thus, a greater emphasis on program implementation of health and nutrition counselling is required to ensure effective uptake of health and nutrition knowledge, and improved dietary and food consumption practices. Our findings emphasize the need for programs that aim to address the gaps in education delivery and counselling at the ANC clinics. Maternal knowledge, attitudes, and practices are important evaluation indicators to assess the impact of educational intervention offered through ANC clinics. Future emphasis on multi-level qualitative and quantitative research in this context may facilitate a greater understanding of determinants of health and nutrition knowledge, attitudes and practices among pregnant women.

## Abbreviations

ANC: Antenatal care; WHO: World Health Organization; HKS: Health knowledge score; NKS: Nutrition knowledge score; AS: Attitude score; DDS: Dietary diversity score; SES: Socio-economic status; ICC: Intra-class correlation.

## Competing interests

There are no competing interests declared other than those identifiable through institutional affiliations.

## Authors’ contributions

NP co-conceived the research, developed the conceptual framework, led statistical analyses and interpretation, wrote the initial manuscript, and iteratively revised the manuscript. DCC co-conceived the research, co-designed the survey, participated in analysis and interpretation, and iteratively revised the manuscript. HZO co-designed the research methods, participated in data acquisition and provided technical and material support. C. Loechl co-conceived and co-designed the research. JL participated in conception and design of the project and participated in project coordination. C. Levin participated in conception and design of the project and provided technical support. KS was involved in development of survey methods, constructed the wealth index and participated in project coordination. CK, JK and MO were involved in acquisition of data and project coordination. All authors read and approved the final manuscript.

## Pre-publication history

The pre-publication history for this paper can be accessed here:

http://www.biomedcentral.com/1471-2393/13/146/prepub

## Supplementary Material

Additional file 1 **Variables used for data analyses.** Variables used from baseline survey to create knowledge, attitudes and practice scores. Click here for file

Additional file 2 **Comparison of intra-class correlations (ICC) to assess between and within community variances in maternal KAP scores.** Assessing the amount of variances due to between and within community differences in the six dependent variables of interest. Click here for file

Additional file 3 **Descriptive statistics for contextual variables, overall and by ANC attendance.** Community level variables collected in the baseline survey pertinent to women’s knowledge, attitudes and practice scores. Click here for file

## Aknowledgements

Funding for this project was provided by the Bill and Melinda Gates Foundation to the International Potato Center. NP received funding from the Canadian Institutes of Health Research. The authors would like to thank the women who took the time to participate in this survey, the enumerators who conducted the interviews as well as the project staff for their efforts in data acquisition.
