# Supplementary material for: Health and nutrition knowledge, attitudes and practices of pregnant women attending and not-attending ANC clinics in Western Kenya: a cross-sectional analysis
Source: BMC Pregnancy Childbirth. 2013 Jul 11;13:146. doi: 10.1186/1471-2393-13-146 (PMC3716969; doi:10.1186/1471-2393-13-146)
Supplement: Additional file 1 — Variables used for data analyses. Variables used from baseline survey to create knowledge, attitudes and practice scores. [file 1471-2393-13-146-S1.docx]

**Table S1:** Survey items used to generate dependent summative scores of nutrition knowledge, health and healthcare knowledge, attitudes, and dietary diversity

| **Outcomes of Interest** | **Dependent Variables** | **Survey items** | **Variable Construction** |
| --- | --- | --- | --- |
| Knowledge Scores | Nutrition Knowledge Score | - Knowledge of three food groups - Main sources of energy - Main source of body-building foods - Main sources of protective foods | Equally-weighted sub-scales per question, and summative score |
|  | Health and healthcare Knowledge Score | - Eating well (good quality foods). - Avoid drinking alcohol - Do not Smoke - Attend ANC-care clinic - Take iron tables or iron syrup - Protect from malaria with nets/drugs - Check HIV Status - Other (Avoid stress/exercise) (equally-weighted sub-scale) - When should a pregnant woman start going to ANC clinic? - How many times to attend ANC - List 3 activities that occur at ANC clinic (equally-weighted sub-scale) | Summative score |
| Attitude Score | Likert Scale | - Importance of dietary diversity (“*Sam is upset with his wife because instead of giving him ugali every day, some days she makes rice, other days sweetpotato, and once a week cassava. These foods do not provide the same energy as ugali. Sam feels it is best to follow tradition and eat ugali every day. Do you agree with Sam?”*) - Understands nutritive value of foods (“*Emily feeds everyone in her household sweetpotato for breakfast because it is more nutritious than bread.*”) - Proper nutrition during pregnancy (“*Dorothy has just found out that she is pregnant. She is going to try and avoid gaining too much weight during her pregnancy because she knows that if the baby is large she will have a difficult delivery.*”) - F09: Decrease excessive workload during last month of pregnancy (“*Poko is in her 9th month of pregnancy. She has had several children before. Every day she goes to the family farm to labor and stay until evening. However, this time, her husband says she should stop cultivating the last month of her pregnancy and just do housework. He will hire some extra labor to help in the field. Poko says the money could be spent on other things. Do you agree with the husband that it is important to cut down on the amount of hard work you do during the last month of pregnancy?*”) - F12: Attending ANC clinic at 6 months is fine (“*Nancy is 4 months pregnant. Her friend Poko is encouraging her to go to the ante-natal clinic. But Nancy says she is busy and usually goes in her 6th month which is soon enough to get all the important treatments needed before the baby comes. Do you agree with Nancy?*”) | Giving higher scores to desired responses; summative score |
| Practices | Dietary Diversity | Household food consumption for 12 groups of foods (1 item per food group type) | Summative score |
|  | Food intake | Eating less, more or the same amount of food during pregnancy | N/A |
|  | Seeking treatment | Taken drugs to prevent malaria and intestinal worms during pregnancy | N/A |
