# Supplementary material for: Health and nutrition knowledge, attitudes and practices of pregnant women attending and not-attending ANC clinics in Western Kenya: a cross-sectional analysis
Source: BMC Pregnancy Childbirth. 2013 Jul 11;13:146. doi: 10.1186/1471-2393-13-146 (PMC3716969; doi:10.1186/1471-2393-13-146)
Supplement: Additional file 2 — Comparison of intra-class correlations (ICC) to assess between and within community variances in maternal KAP scores. Assessing the amount of variances due to between and within community differences in the six dependent variables of interest. [file 1471-2393-13-146-S2.docx]

**Table S1:** Comparison of intra-class correlations (ICC) to assess between and within community variances in maternal KAP scores

| **Variable** | **Variance*** | | | | **ICC** |
| --- | --- | --- | --- | --- | --- |
|  | **Between** | **p-value^1^** | **Within** | **p-value^1^** |  |
| **Nutrition knowledge score** | 0.062 | 0.1491 | 3.703 | <0.0001 | 0.016 |
| **Health knowledge score** | 0 | - | 3.023 | <0.0001 | 0 |
| **Dietary diversity score** | 0.139 | 0.0046 | 1.899 | <0.0001 | 0.068 |
| **Attitude score** | 0.232 | 0.0150 | 4.537 | <0.0001 | 0.049 |

*Total variance for dependent variables is dichotomized into between (between villages) and within (participants within village) variances.

^1^ p-values for estimates are significant at an alpha <=0.05
