# Supplementary material for: Health and nutrition knowledge, attitudes and practices of pregnant women attending and not-attending ANC clinics in Western Kenya: a cross-sectional analysis
Source: BMC Pregnancy Childbirth. 2013 Jul 11;13:146. doi: 10.1186/1471-2393-13-146 (PMC3716969; doi:10.1186/1471-2393-13-146)
Supplement: Additional file 3 — Descriptive statistics for contextual variables, overall and by ANC attendance. Community level variables collected in the baseline survey pertinent to women’s knowledge, attitudes and practice scores. [file 1471-2393-13-146-S3.docx]

**Table S1:** Descriptive statistics for village-level variables, overall and by ANC attendance

| **Variable** | **Overall** | **ANC Attendance^a^** | | **p-values*** |
| --- | --- | --- | --- | --- |
|  |  | **Attending** | **Non-attending** |  |
| **n (%)** | 979 | 582 (59.4) | 382 (39.0) |  |
| **Village size, households** |  |  |  |  |
| Mean ± SD | 142.47 ± 51.7 | 140.95 ± 51.7 | 144.67 ± 51.5 | 0.2746 |
| Median | 142 | 139 | 144 |  |
| Range | [38, 296] | 250 | 258 |  |
| **Health meetings in community, yes^b^** | 642 (72.5) | 374 (71.8) | 257 (73.2) | 0.6423 |
| **Village health/nutrition committee, yes^b^** | 618 (69.8) | 357 (68.5) | 250 (71.2) | 0.3947 |
| **Other health interventions in village, yes^b^** | 450 (50.8) | 260 (49.9) | 181 (51.6) | 0.6300 |
| **Distance to health facility, minutes^c^** |  |  |  |  |
| Mean ± SD | - | 41.29 ± 32.8 | - | - |
| Median | - | 30.0 | - | - |
| Range | - | [2, 180] | - | - |

*p-values for Student’s t-test and Chi-square test for comparison of means and proportions are significant at a two-sided alpha of <0.05

^a^ Missing ANC attendance data for n=15 women, effecting sample size for ANC attendance comparisons, n=964;

^b^ Missing values n=93 women since community level data missing for n=7 villages; effective n=886 women.

^c^ Distance in minutes to health facility by walking, by bicycle or by car. Only available for women who attend ANC clinics
